# Analysis of bronchial and vascular patterns in left upper lobes to explore the genesis of mediastinal lingular artery and its influence on pulmonary anatomical variation

**DOI:** 10.1186/s13019-021-01682-w

**Published:** 2021-10-18

**Authors:** Chuan Gao, Wen-zheng Xu, Zhi-hua Li, Liang Chen

**Affiliations:** 1 Department of Cardiothoracic Surgery, Jinling Hospital, Nanjing, Jiangsu China; 2 Department of Thoracic Surgery, Jiangsu Province People’s Hospital and the First Affiliated Hospital of Nanjing Medical University, Nanjing, 210029 Jiangsu China

**Keywords:** Mediastinal lingular artery (MLA), Left upper lobe (LUL), Lung development, Anatomical variation, Three-dimensional computed tomography bronchography and angiography (3-DCTBA)

## Abstract

**Background:**

For thoracic surgeons, three-dimensional computed tomography bronchography and angiography (3-DCTBA) is a convenient way to analyze pulmonary variations before segmentectomy. Mediastinal lingular artery (MLA) is one of the representative variations.

**Methods:**

The 3-DCTBA data of left upper lobe (LUL) were collected from patients who underwent pulmonary surgery from January 2018 to December 2019. We reviewed the patterns of bronchi and pulmonary vessels and grouped them according to different classifications.

**Results:**

Among all the 404 cases of 3-DCTBA, mediastinal lingular artery (MLA) was found in 107 cases (26.49%). The patterns of B^3^ and the vein in left upper division (LUD) are distinct between mediastinal (M-type) group and interlobar (IL-type) group. The patterns of bronchi and veins in lingular division, as well as the pattern of pulmonary artery in LUD, have no differences between M-type and IL-type groups.

**Conclusions:**

Mediastinal lingular artery is speculated to originate from the variation of B^3^, and the MLA independently influences the venous pattern in LUD in turn.

## Introduction

The development of three-dimensional computed tomography bronchography and angiography (3-DCTBA) imaging brings convenience for thoracic surgeons to know the precise anatomical relationship among bronchi, pulmonary artery, and vein before pulmonary surgery [1–3]. As increasing reports about the variations of pulmonary anatomy in 3-DCTBA have been published [4–7], how to understand the complex and diverse variations becomes desirable.

In previous studies on pulmonary morphology, the objects are mainly human embryo specimens [8–10], which brought direct evidence for the origins of some congenital anomalies and variations. Meanwhile, given the discrepancies of pulmonary morphology between embryo and adult, these researches still can't clarify the common variations that thoracic surgeons often meet during pulmonary surgery. Now accumulating mass of the 3-DCTBA data from adults make it possible to study these anatomical variations.

We chose the mediastinal lingular artery (MLA), one of the most common variations [11, 12], as the study object, and analyzed the different patterns of bronchi and vessels in left upper lobe (LUL) by categorizing them into mediastinal (M-type) group and interlobar (IL-type) group. In this way, this study aims to explore the genesis of MLA and provide a perspective to understand pulmonary anatomical variations in left upper lobe influenced by MLA.

## Materials and methods

### Patients and 3-DCTBA models

Patients in our department routinely received contrast-enhanced computed tomography test (Siemens 64-slice dual-source CT) before surgery and most CT images with solid pulmonary nodules (SPNs), and all of the images with ground-glass nodules (GGNs) would be constructed by Deepinsight platform (Neusoft Group Ltd.) into three-dimensional computed tomography bronchography and angiography(3-DCTAB). Our study collected the 3-DCTBA data of left upper lobe (LUL)from patients who underwent pulmonary surgery from January 2018 to December 2019. The 3D models containing bronchi and pulmonary vessels in each case, sometimes combining with the primitive CT images, were repeatedly inspected and sorted according to different classification standards by 2 thoracic surgeons. This study was approved by the ethics committee of the Jiangsu Province People's Hospital, and informed consent was obtained from each patient.

### Classification of pulmonary vessels and bronchi in the left upper lobe (LUL)

**Fig. 1:**
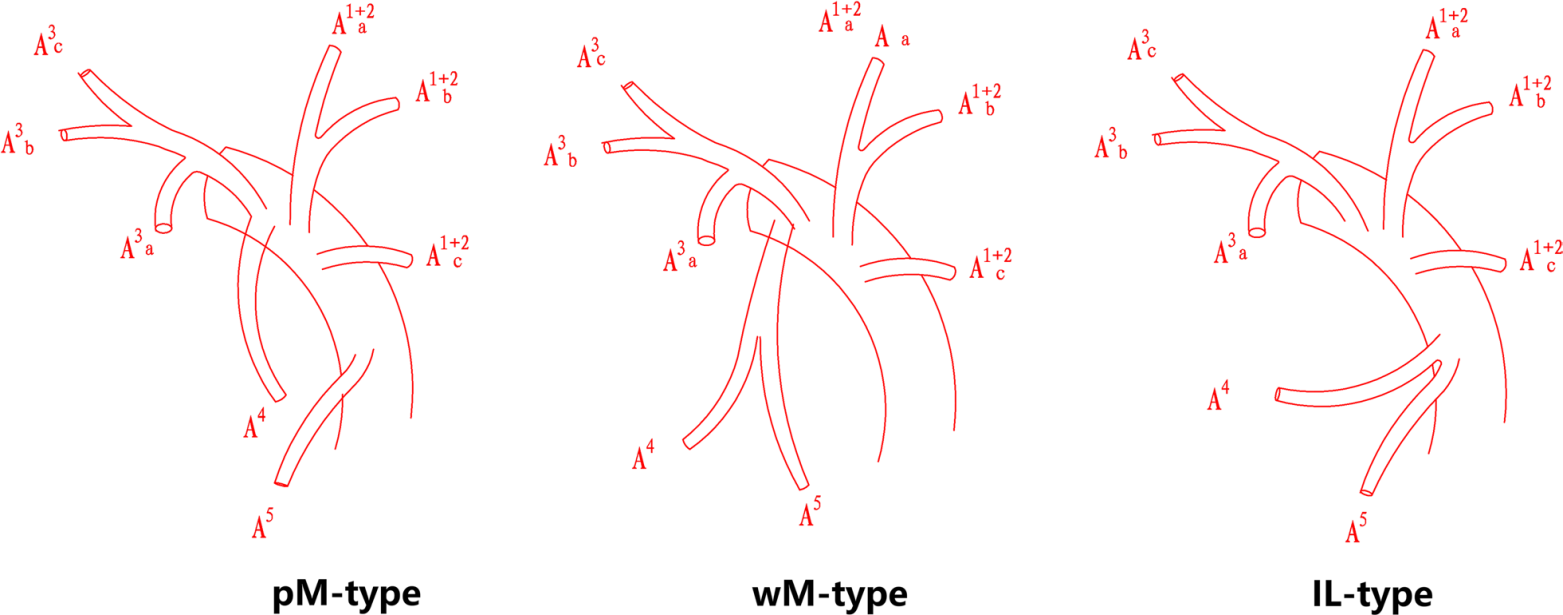
Type of lingular artery according to whether the lingular artery comes from the mediastinal or interlobar part of the pulmonary trunk

**Fig. 2:**
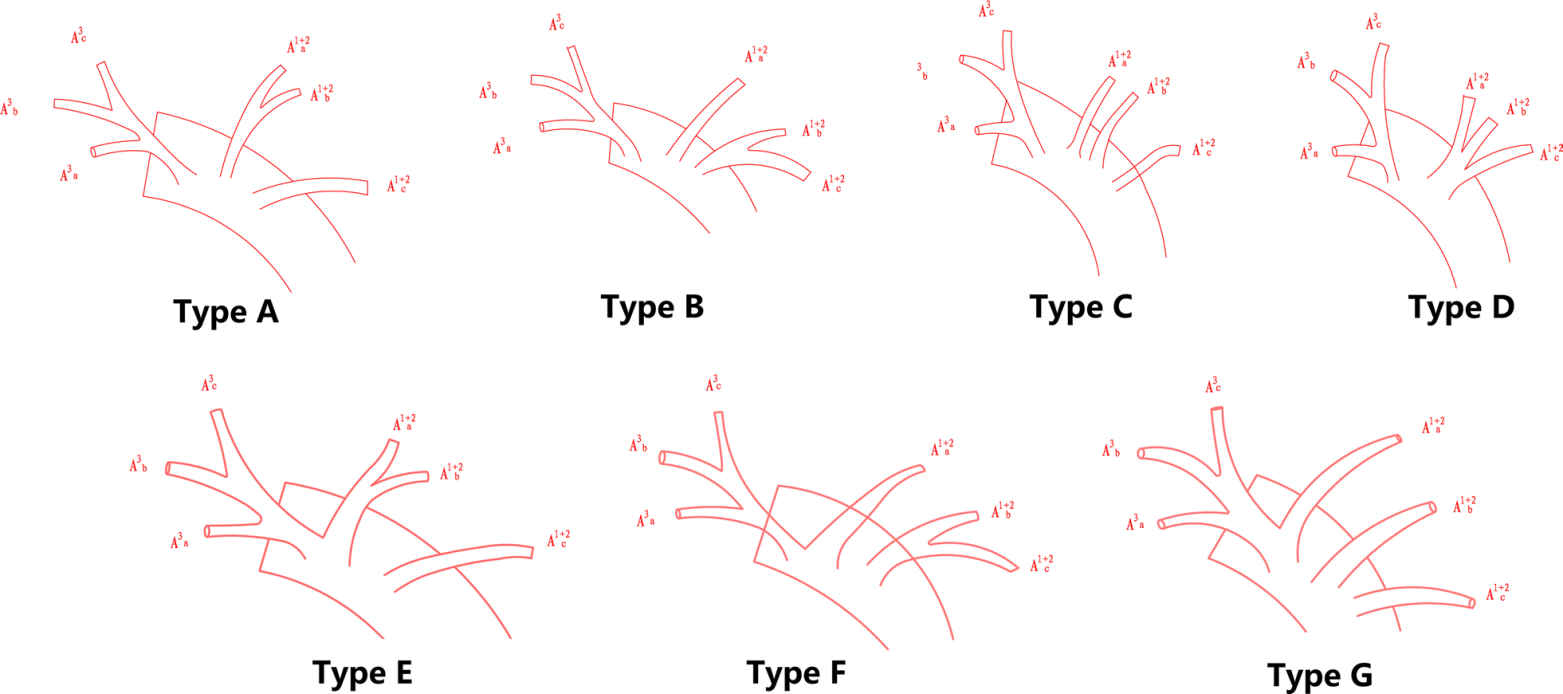
When A^3^ is independent with A^1+2^, there are four types: Type A (A^1+2^a + b, A^1+2^c); Type B (A^1+2^a, A^1+2^b + c); Type C (A^1+2^a, A^1+2^b, A^1+2^c); Type D (A^1+2^abc); When A^3^ and A^1+2^ have common trunk, there are three types: Type E (A^3^ + A^1+2^a + b, A^1+2^c); Type F (A^3^ + A^1+2^a, A^1+2^b + c);Type G (A^3^ + A^1+2^a, A^1+2^b, A^1+2^c)

According to whether the lingular artery comes from the mediastinal or interlobar part of the pulmonary trunk, the lingular artery of LUL was categorized into mediastinal type (M-type) and interlobar type (IL-type). Mediastinal type is further divided into partially mediastinal type(pM-type) and wholly mediastinal type(wM-type) (Fig. 1). apicoposterior + anterior artery (A^1+2^ + A^3^) artery has 7 types (Fig. 2) referring to Maciejewski classification method [13]. A^3^ is independent with A^1+2^: Type A (A^1+2^a + b, A^1+2^c); Type B (A^1+2^a, A^1+2^b + c); Type C (A^1+2^a, A^1+2^b, A^1+2^c); Type D (A^1+2^abc); A3 has common trunk with A^1+2^: Type E (A^3^ + A^1+2^a + b, A^1+2^c); Type F (A^3^ + A^1+2^a, A^1+2^b + c); Type G (A^3^ + A^1+2^a, A^1+2^b, A^1+2^c). It should be noted in this classification that A^3^ means the segmental trunk because subsegmental branch such as A^3^b or A^3^c may ramify from lingular artery or left pulmonary artery in M-type or other rare variations.

**Fig. 3:**
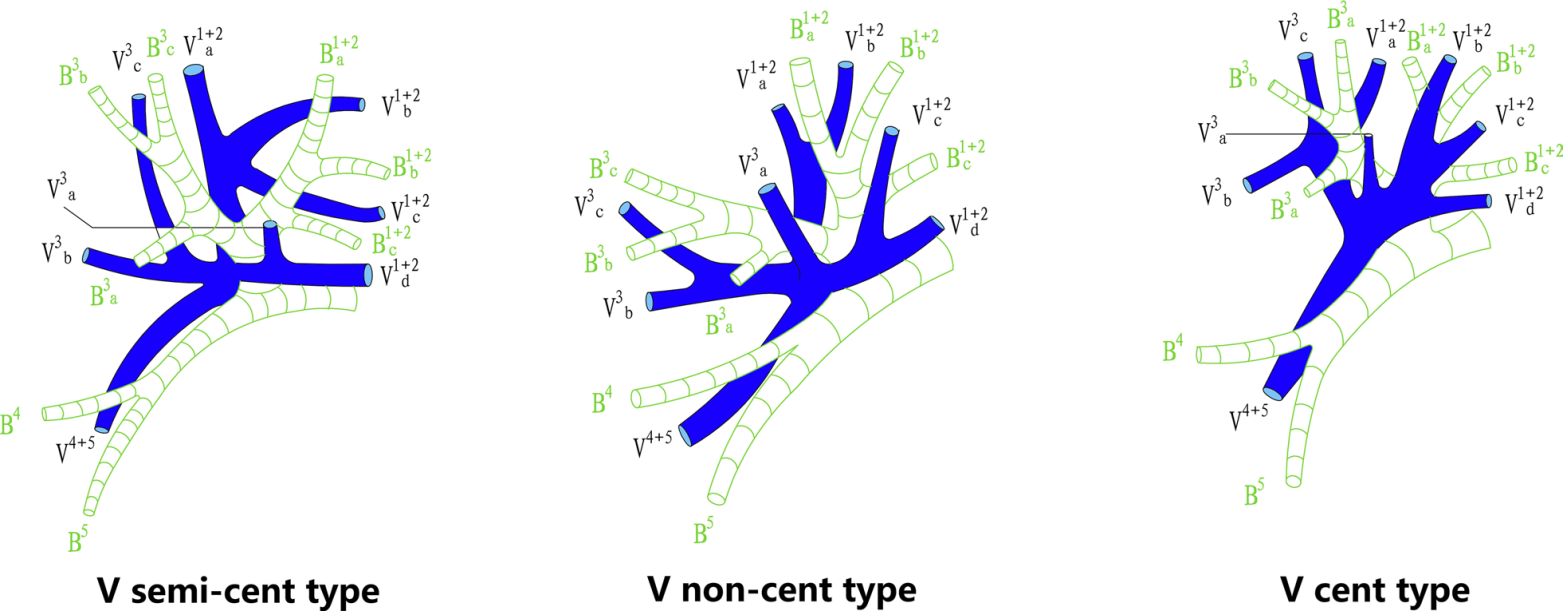
Patterns of pulmonary veins in left upper division according to Yamashita's description and the nomenclature

**Fig. 4:**
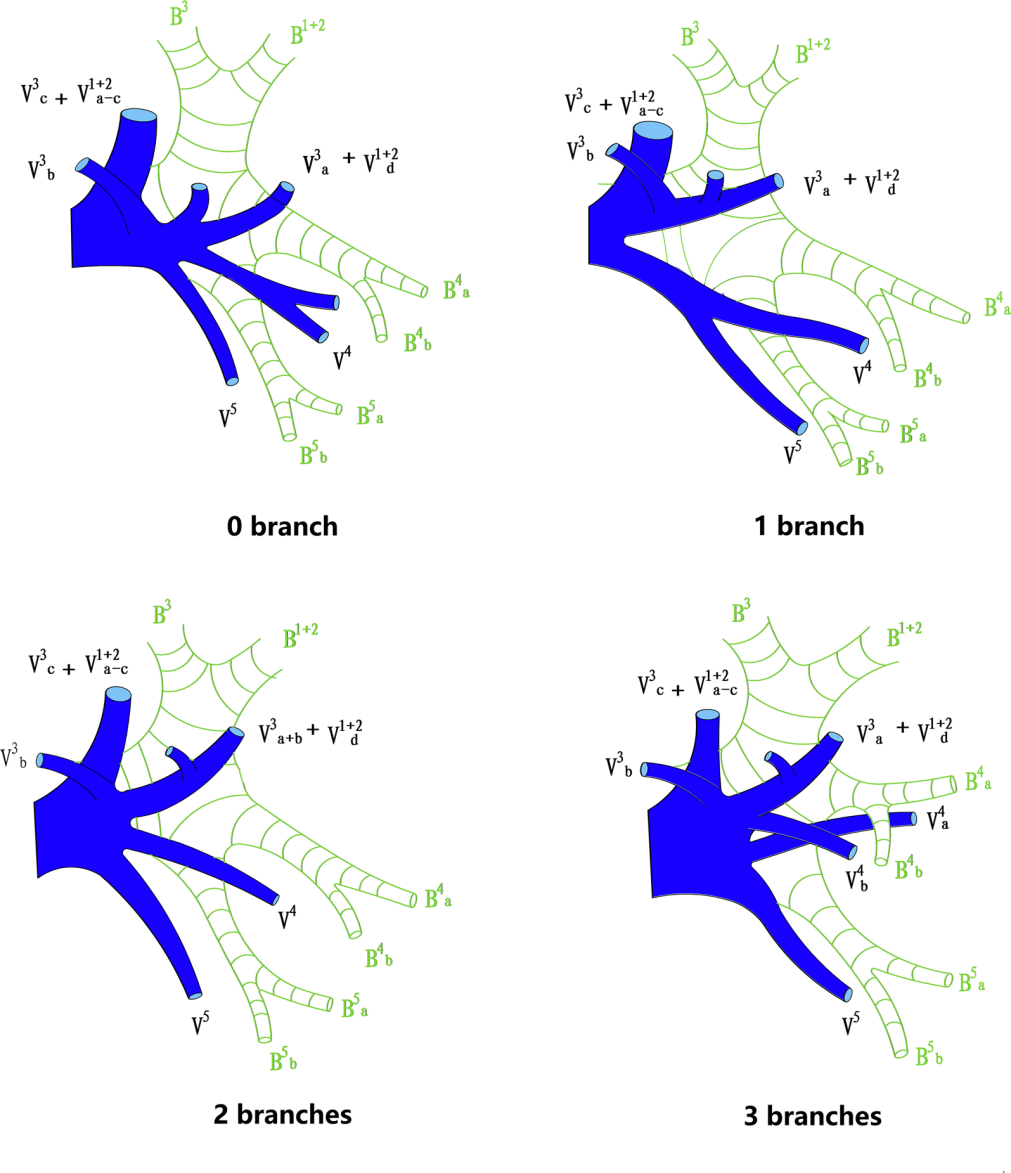
Type of lingular vein according to the number of independent intrasegmental veins

According to Yamashita's description and the nomenclature of venous pattern in LUL, patterns of pulmonary veins in left upper division (LUD) are divided into three types [14]. (1) semi-central vein type (V semi-cent): V^1+2^a, V^1+2^b, V^1+2^c (sometimes including V^1+2^d) forms a common trunk and drains into left upper pulmonary vein (LUPV)above B^3^ on the mediastinal side; (2) central vein type (V cent): V^1+2^b (sometimes including V^1+2^a), V^1+2^c and V^1+2^d drain together before they join with V^3^b below B^3^ and finally flow into LUPV. (3) noncentral vein type (V non-cent): V^1+2^a and V^1+2^b drain cephalad into the LUPV above B^3^, and V^1+2^c and V^1+2^d drain caudally into V^3^a and V^3^b below B^3^ before they flow into LUPV (Fig. 3). The lingular vein is sorted according to the number of independent intrasegmental veins (0 to 3 branches) (Fig. 4).

**Fig. 5:**
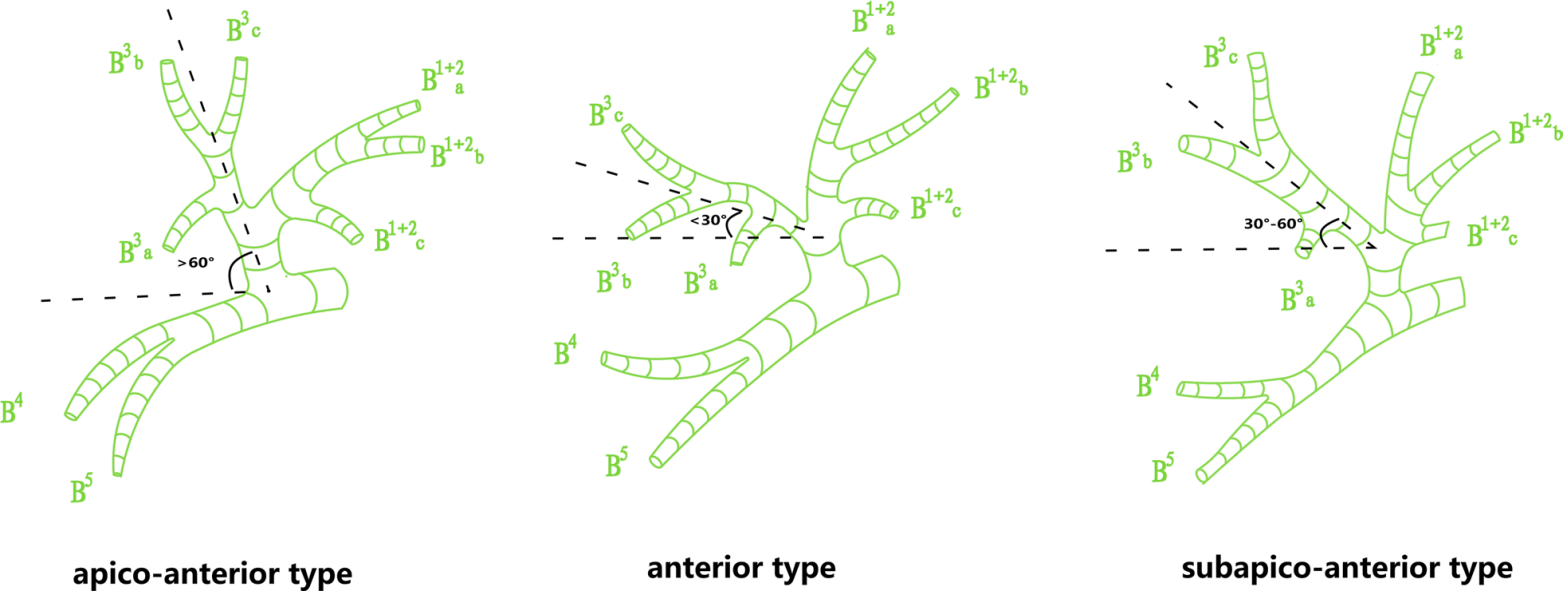
Classification of B^3^ patterns according to the angle between the horizontal line and the central line of B^3^ bronchus

**Fig. 6:**
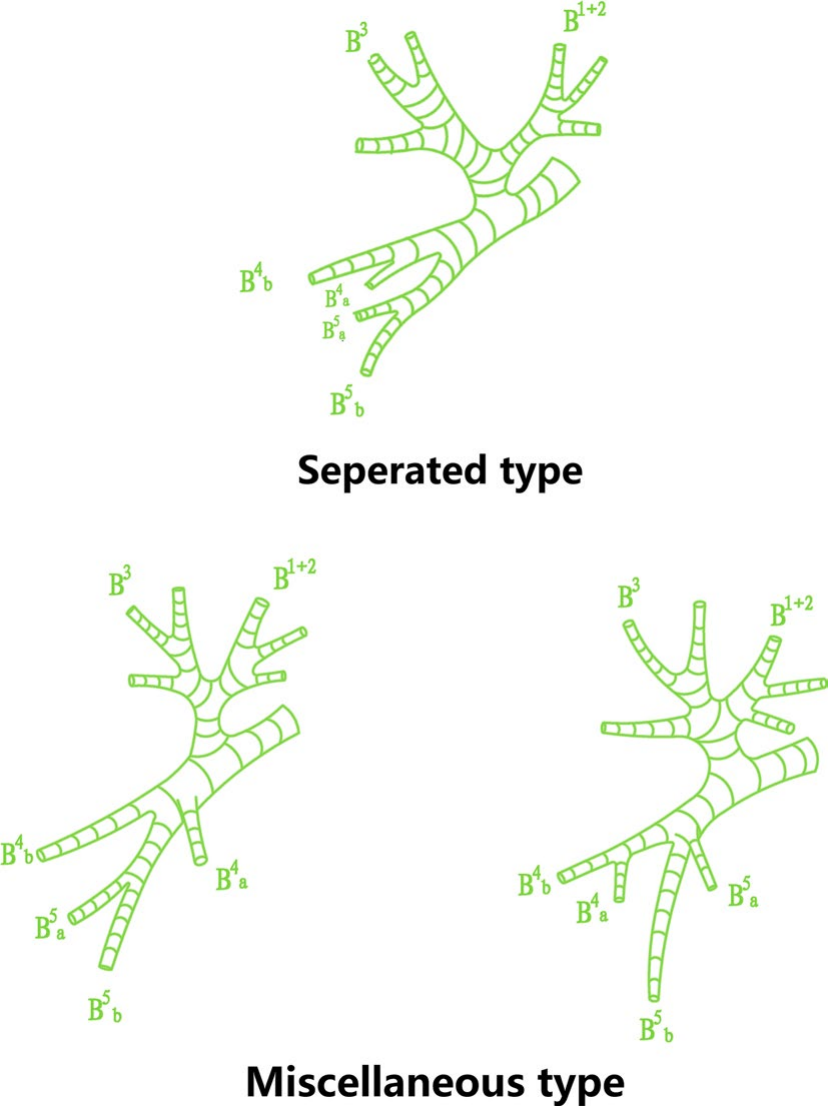
Type of lingular bronchus according to whether B^4^ and B^5^ are independent

Classification of B^3^ patterns was defined according to the angle between the horizontal line and the central line of B^3^ bronchus referring to Yamashita’s description and nomenclature methods [5, 14] (Fig. 5): anterior type: less than 30°, subapicoanterior type: between 30° and 60°, apico-anterior type: more than 60°. Lingular bronchus was sorted into 2 types: separated type of which B^4^ and B^5^ are independent, and miscellaneous type of which B^4^ or B^5^ could not be classified into two independent branches (Fig. 6).

### Data collection and Statistical analysis

Bronchi and pulmonary vessels in LUL were inspected with help of the 3D-model image and different patterns were categorized and recorded into a database. Statistical analysis was performed using SPSS 19.0 software (SPSS Inc, Chicago, IL). Categoric variables were compared using Pearson Chi-Square test, and the test is valid when no more than 20% of the expected counts are less than 5 and all individual expected counts are 1 or greater. A *P* value less than 0.05 is considered statistically significant.

## Results

### Patterns and distribution of bronchi and pulmonary vessels in LUL

#### Branching patterns of the pulmonary artery

Quantitatively, IL-type were found in 297 cases (73.51%) and M-type were found in 107 cases (26.49%), among which 84 cases (20.79%) belong to pM-type and 23 cases (5.69%) belong to wM-type. For Apicoposterior + anterior(A^1+2^ + A^3^) artery, Type A were found in 109 cases (26.98%), Type B in 28 cases (5.93%), Type C in 31 cases (7.67%), Type D in 53 cases (13.12%), Type E in 49 cases (12.13%), Type F in 68 cases (16.83%), Type G in 66 cases (16.34%).

#### Branching patterns of the pulmonary vein

Among different patterns of pulmonary vein in LUD, V semi-cent type were found in 254 patients (62.87%); V cent type in 114 patients (28.22%); V non-cent type in 36 patients (8.91%). The independent intrasegmental lingular vein were found 0 branch in 7 cases (1.73%), 1 branch in 245 cases (60.64%), 2 branches in 66 cases (16.34%) and 3 branches in 1 case (0.25%).

#### Branching patterns of the bronchi

For patterns of B^3^ bronchus, 111 cases (27.48%) were classified into anterior type, 104 cases (25.74%) into apico-anterior type, and 189 cases (46.78%) into subapicoanterior type. As for lingular bronchus, separated types were found in 299 cases (74.01%) and miscellaneous types were found in 105 cases (25.99%).

### Distribution of different patterns between IL-type and M-type group (wM-type and pM-type)

As detailed in Table 1, there were 50 cases (12.38%) with subapico-anterior bronchial type, 46 cases (11.39%) with apico-anterior type, 11 cases (2.73%) with anterior type in the M-type group, and 139 cases (34.41%) with subapico-anterior bronchial type, 58 cases (14.36%) with subapico-anterior type, 100 cases (24.75%) with anterior type in the IL-type group. This indicated that the apico-anterior bronchial type was more common in the M-type group, while the anterior bronchial type was more common in the IL-type group (*P* < 0.001).

**Table 1:** Distribution of branching patterns in left upper division between different lingular artery types

	wM-type	pM-type	*P* value	M-type	IL-type	total	*P* value
*Bronchial type in upper division*							
Subapico-anterior type	13 (3.22%)	37 (9.16%)	*P* = 0.568	50 (12.38%)	139 (34.41%)	189 (46.78%)	*P* < 0.001
Apico-anterior type	8 (1.98%)	38 (9.41%)		46 (11.39%)	58 (14.36%)	104 (25.74%)	
Anterior type	2 (0.5%)	9 (2.23%)		11 (2.73%)	100 (24.75%)	111 (27.48%)	
*PV type in upper division*							
Semi-cent type	10 (2.48%)	33 (8.17%)	*P* = 0.793	43 (10.64%)	211 (52.23%)	254 (62.87%)	*P* < 0.001
Non-cent type	1 (0.25%)	7 (1.73%)		8 (1.98%)	28 (6.93%)	36 (8.91%)	
Cent type	12 (2.97%)	44 (10.89%)		56 (13.86%)	58 (14.36%)	114 (28.22%)	
*Apicoanterior + posterior artery type*							
Type A	7 (1.73%)	19 (4.70%)	N.E.^*^	26 (6.44%)	83 (20.54%)	109 (26.98%)	*P* = 0.192
Type B	4 (0.99%)	6 (1.49%)		10 (2.48%)	18 (4.46%)	28 (6.93%)	
Type C	2 (0.50%)	4 (0.99%)		6 (1.49%)	25 (6.19%)	31 (7.67%)	
Type D	4 (0.99%)	16 (3.96%)		20 (4.95%)	33 (8.17%)	53 (13.12%)	
Type E	1 (0.25%)	8 (1.98%)		9 (2.23%)	40 (9.90%)	49 (12.13%)	
Type F	2 (0.5%)	19 (4.7%)		21 (5.20%)	47 (11.63%)	68 (16.83%)	
Type G	3 (0.74%)	12(2.97%)		15 (3.71%)	51 (12.62%)	66 (16.34%)	
*Lingular bronchial type*							
Separated type	17 (4.21%)	59 (14.60%)	*P* = 0.932	76 (18.81%)	223 (55.20%)	299 (74.01%)	*P* = 0.238
Miscellaneous type	6 (1.49%)	25 (6.19%)		31 (7.67%)	74 (18.32%)	105 (25.99%)	
*Independent intrasegmental lingular vein*							
0 branch	0	0	*P* = 0.654^#^	0	7 (1.73%)	7 (1.73%)	*P* = 0.192^#^
1 branch	17 (4.21%)	68 (16.83%)		85 (21.03%)	245 (60.64%)	330 (81.67%)	
2 branches	6 (1.49%)	15 (3.71%)		21 (5.2%)	45 (11.14%)	66 (16.34%)	
3 branches	0 (0%)	1 (0.25%)		1 (0.25%)	0	1 (0.25%)	
Total	23 (5.69%)	84 (20.79%)		107 (26.49%)	297 (73.51%)	404 (100%)	

*50% cells of the expected counts are less than 5 and the minimum expected counts is 1.29

#As more than 20% cells of the expected counts are less than 5 and some minimum expected counts are less than 1,when received Chi Square test, "0 branches" and "1 branch","2 branches" and "3 branches" are grouped together

The semi-cent vein type, the non-cent type, and the cent type accounted for 43(10.64%), 8(1.98%), 56(13.86%) in the M-type group, and 211(52.23%), 28(6.93%), 58(14.36%) in the IL-type group respectively. It was found that the semi-cent type was often combined with IL-type, and cent type frequently appeared in the M-type group (*P* < 0.001). In further examination, we made the Cochran–Mantel–Haenszel (CMH) chi-square test to determine the independent influence factors on the PV (pulmonary vein) type in LUD. As shown in Table 2, MLA had an independently significant influence on PV type in subapico-anterior bronchial group (*P* < 0.001) and apico-anterior type group (*P* = 0.01) respectively. In anterior bronchial group, the Chi-Square test was invalid as more than 20% of the expected counts were less than 5 and the minimum individual expected count was less than 1. Meanwhile, the bronchial type in LUD was also an independent factor on PV type in IL-type group (*P* < 0.001). In M-type group, the Chi-Square test was invalid as more than 20% of the expected counts were less than 5 and the minimum individual expected count was less than 1.

**Table 2:** Types of PV among different types of bronchus and pulmonary artery in left upper division

Bronchial type in upper division	Pulmonary artery type	PV type in upper division			Total	*P* value
		Semi-cent type	Non-cent type	Cent type		
Subapico-anterior type	M-type	22 (5.45%)	3 (0.74%)	26 (6.44%)	51 (12.62%)	*P* < 0.001
	IL-type	97 (24.01%)	14 (3.47%)	27 (6.68%)	138 (34.16%)	
Apico-anterior type	M-type	10 (2.48%)	5 (1.24%)	30 (7.43%)	45 (11.14%)	*P* = 0.01
	IL-type	30 (7.43%)	6 (1.49%)	23 (5.69%)	59 (14.60%)	
Anterior type	M-type	11 (2.72%)	0	0	11 (2.72%)	N.E.^*^
	IL-type	84 (20.79%)	8 (1.98%)	8 (1.98%)	100 (24.75%)	
Subapico-anterior type	M-type	22 (5.45%)	3 (0.74%)	26 (6.44%)	51 (12.62%)	*N.E.*^#^
Apico-anterior type		10 (2.48%)	5 (1.24%)	30 (7.43%)	45 (11.14%)	
Anterior type		11 (2.72%)	0	0	11 (2.72%)	
Subapico-anterior type	IL-type	97 (24.01%)	14 (3.47%)	27 (6.68%)	138 (34.16%)	*P* < 0.001
Apico-anterior type		30 (7.43%)	6 (1.49%)	23 (5.69%)	59 (14.60%)	
Anterior type		84 (20.79%)	8 (1.98%)	8 (1.98%)	100 (24.75%)	

*33.3% cells of the expected counts are less than 5 and the minimum expected counts is 0.79

^#^44.4% cells of the expected counts are less than 5 and the minimum expected counts is 0.82

In regards to the type of apicoposterior + anterior artery (A^1+2^ + A^3^), there were 26 cases (6.44%) of type A, 10 cases (2.48%) of type B, 6 cases (1.49%) of type C, 20 cases (4.95%) of type D, 9 cases (2.23%) of type E, 21 cases (5.2%) of type F, and 15 cases (3.71%) of type G in the M-type group. The according type accounted for 109(26.98%), 28(6.93%), 31(7.67%) 53(13.12%), 49(12.13%), 68(16.83%), 66(16.34%) respectively in the IL-type group. There was no significant difference between M-type group and IL-type group on the type of apicoposterior + anterior artery (A^1+2^ + A^3^) (*P* = 0.192). The separated and miscellaneous type of lingular bronchus was 76(18.81%) and 31(7.67%) in the M-type group, 223(55.20%) and 74(18.32%) in the IL-type group. No significant difference was found between IL-type and M-type group (*P* = 0.238). The number of independent intrasegmental lingular vein in M-type and IL-type group with 0 branch were 0 and 7 (1.73%), 1 branch 85(21.03%) and 245(60.64%), 2 branch 21(5.2%) and 45(11.14%), 3 branch 1(0.25%) and 0. As more than 20% cells of the expected counts were less than 5 and some minimum expected counts were less than 1, "0 branches" and "1 branch","2 branches" and "3 branches" were grouped together when received Chi Square test. Consequently, M-type group and IL-type group didn’t have difference either (*P* = 0.192).

When we compared the corresponding types of bronchus and vessels between wM-type and pM-type groups, there was no significant difference observed (*P* > 0.05). The detailed results were recorded in Table 1.

## Discussion

Among all the variations in both lungs, the mediastinal lingular artery (MLA) may be the most common and distinguishable one which doesn’t parallel the accompanying bronchi at proximal [15]. In our study, we choose MLA as layered strata to analyze the different patterns of bronchi and vessels between M-type and IL-type group. By discussing the distribution of patterns respectively, we try to provide a perspective to understand the genesis of MLA and its influence on pulmonary anatomy in left upper lobe (LUL).

The variations and aberrant branches of lung originate from the complex development of the embryo. According to DeMello’s study [16], the formation of vascular tree contains two mechanisms: angiogenesis and vasculogenesis. Angiogenesis produces the proximal central trunks by branching of new vessels from preexisting ones, and vasculogenesis produces a distal capillary network by the development of blood lakes that transform to vessels. Thus, it is reasonable to speculate that the MLA originates from angiogenesis. For the development of human lung, the timeline is described as that airway emerges before pulmonary artery and vein appears afterward [17, 18]. Hall confirmed that bronchus is necessary for pulmonary artery during the development stage, as smooth muscle from the airway makes up the innermost layers of pulmonary artery when it becomes mature [19], whereas vasculature is dispensable for epithelial branching of bronchi at its embryonic development stage [20, 21].

Combined with our results, the B^3^ bronchial patterns seem to play an important part in the formation of MLA. Metzger et al. studied the early bronchial tree by examining chemically fixed lung tissue from mouse embryos using microscopy. They have revealed that bronchi branching in three geometrical modes: domain branching, planar bifurcation, and orthogonal bifurcation [22]. These modes reasonably explain the different patterns of B^3^. One of the reasons why the different patterns of B^3^ are relevant to the probability of MLA, from our perspective, is that the apico-anterior type of B^3^ leaves more space for lingular bronchus by the side of hilus, generating artery easily from left pulmonary trunk into lingular division. Another concept assumed by Onuki et al. [23] that ‘the lung segments never continuously exist from the early stage of the embryonic period as units, but they are only simple units artificially named by their prevailing bronchial branching patterns’. They also found that the combination of anterior extension type of B^3^ bronchus with the inter-lobar type (IL-type) of arterial branching was often observed, as well as the combination of apico-anterior extension type with the mediastinal type (M-type) [7]. They speculated the location, which originally would be S^3^ in IL-type, would become a part of S^4+5^ in M-type after the whole axis of LUL rotates.

We also verified that the distribution of bronchial and vascular branching patterns in LUL between wM-type and pM-type has no significant difference. This implies that wM-type and pM-type may result from similar embryonic development.

Our layered testing results revealed that the distribution of PV type in LUD is distinct among the three different patterns of B^3^ and between M-type and IL-type groups. As demonstrated in the 3-DCTBA, pulmonary vein lies at a distance from bronchus, suggesting that pulmonary vein, different from pulmonary artery which received muscle cells from bronchus, is influenced less by airway. However, Hall [18] has used immunohistochemical techniques to study serial sections of human embryonic and fetal lungs. And his study indicated that veins ran midway between airways where the mesenchymal cell density was low. In other words, the bronchial pattern shapes the density of mesenchymal cells during embryonic stage which further influences the pattern of pulmonary vein. Interestingly, when excluded the influence from the patterns of B^3^, the venous pattern of LUD is still affected by the MLA independently. Dejima et al. [11] have concluded that MLA is associated with greater artery diameter and larger lingular division volume. This indicates that the lingular division in M-type group has more blood flow volume. Our study revealed that V cent type vein often appears in M-type group and V semi-cent type often appears in IL-type group. Whether the different venous patterns reveal not only the variations of pulmonary vein in LUD, but also the blood flow distribution in LUL? This relationship between different branching patterns of PV and the blood flow distribution in LUL needs to be investigated by precise data and appropriate analytical methods.

As the apicoposterior + anterior artery (A^1+2^ + A^3^) of LUL is characterized by its greatest variability, we used the classification proposed by Boyden and Hartman [24] in 1946 to explore whether these subtypes have a significant influence on the genesis of MLA. Our study revealed that the distribution of these artery subtypes had no difference between M-type and IL-type groups, indicating that the pattern of pulmonary artery in LUD is irrelevant with the generation of MLA. In previous research or anatomical atlas [24–26], lingular bronchi are generally divided into B4 and B5. However, based on our observation, 25.99% of lingular bronchi belong to miscellaneous type which means B^4^ and B^5^ are not independent. The different lingular bronchial patterns seem to have little influence on the formation of MLA as there is no significant difference in lingular bronchial patterns between M-type and IL-type groups. Besides, inspired by Dejima’s study about the blood flow of lingular division in M-type group [11], we also counted the ramies of independent intrasegmental lingular vein, but there is no distinction between M-type and IL-type group even though the number in M-type seems to exceed that in IL-type.

This present study has possible limitations. First, the relation of bronchus and vessels demonstrated by 3-DCTBA may reverse causation, or even be interpreted as the false causation, because the patterns of bronchi and vessels in M-type group can’t certify embryonic development directly. Second, the classifications of bronchi and pulmonary vessels are empirical which may not reflect all the differences objectively. Furthermore, all the 3-DCTBA digital data come from patients undergoing pulmonary surgery, which may introduce selection bias. However, we carried a comprehensive comparison in patterns of bronchi and pulmonary vessels between M-type and IL-type group with a large number of cases, which illustrated the characteristics of pulmonary anatomy in LUL with or without MLA.

## Conclusion

In summary, based on our observation, we proposed that the MLA is originated from the variation of B^3^, and the MLA independently influences the branching pattern of PV in LUD in turn. The different types of MLA (pM-type and wM-type) may come from the similar development process. The influence of MLA on the pattern of pulmonary artery in LUD and of bronchi and pulmonary veins in lingular division was not distinct.

## Data Availability

Please contact author for data requests.

## Abbreviations

3-DCTBA: Three-dimensional computed tomography bronchography and angiography
MLA: Mediastinal lingular artery
LUL: Left upper lobe
LUD: Left upper division
M-type: Mediastinal type of lingular artery
pM-type: Partially mediastinal type of lingular artery
wM-type: Wholly mediastinal type of lingular artery
IL-type: Interlobar type of lingular artery
SPNs: Solid pulmonary nodules
GGNs: Ground-glass nodules
CT: Computed tomography
LUPV: Left upper pulmonary vein
PV: Pulmonary vein
V semi-cent: Semi-central vein type
V cent: Central vein type
V non-cent: Noncentral vein type
